# Postcolectomy Enteritis in a Pediatric Patient With Ulcerative Colitis

**DOI:** 10.1097/PG9.0000000000000255

**Published:** 2022-10-20

**Authors:** Kathryn Hawa, Ross Maltz, Amy Donegan, Vinay Prasad, Jennifer L. Dotson, Brendan Boyle, Hilary K. Michel

**Affiliations:** * Division of Pediatric Gastroenterology, Hepatology and Nutrition, Nationwide Children’s Hospital, Columbus, OH; † Department of Pediatrics, The Ohio State University College of Medicine, Columbus, OH; ‡ Department of Pathology, Nationwide Children’s Hospital, Columbus, OH; § Center for Child Health Equity and Outcomes Research, The Abigail Wexner Research Institute, Nationwide Children’s Hospital, Columbus, OH.

**Keywords:** postcolectomy enteritis, small bowel inflammation, ulcerative colitis, surgical complications

## Abstract

Postcolectomy enteritis is characterized by diffuse small bowel inflammatory changes following colectomy for medically refractory ulcerative colitis. Symptoms may include abdominal pain, massive intestinal bleeding, intestinal perforation, and high stoma output. While the exact pathogenesis is unknown, immune dysregulation with increased cytokine and inflammatory cell response is suspected to lead to the inflammatory response. Therefore, immunosuppressive medications are the mainstay for treatment. All cases to date have been described in adult patients. We present a case of postcolectomy enteritis in a pediatric patient who improved without significant intervention.

## INTRODUCTION

Ulcerative colitis (UC) is characterized by diffuse mucosal inflammation typically diagnosed via colonoscopy (1). In severe disease or when medical therapy fails to manage symptoms, surgical interventions, including total abdominal colectomy, may be indicated (2). A rare postsurgical complication is postcolectomy enteritis (PCE) syndrome, which may present days to months after surgery and is characterized by diffuse inflammation of the small intestine. Presenting signs and symptoms vary, but may include abdominal pain, high ostomy output, severe intestinal bleeding, hypovolemic shock, and disseminated intravascular coagulation. Presentation is differentiated from Crohn’s disease based on timing, histology and diffuse pattern of mucosal involvement (3). The pathogenesis of PCE is unknown, though it is thought to be due to immune dysregulation with increased cytokine and inflammatory cell response. Prior cases have been managed with systemic corticosteroids and antitumor necrosis factor alpha (anti-TNFa) medications (3–7). In the adult literature, Horio et al (6) found the incidence of PCE to be around 0.8%. No cases of PCE are reported in pediatric patients, so little is known about presentation, natural history and outcomes. We present the evaluation and management of a pediatric patient with PCE.

## CASE

**FIGURE 1.**
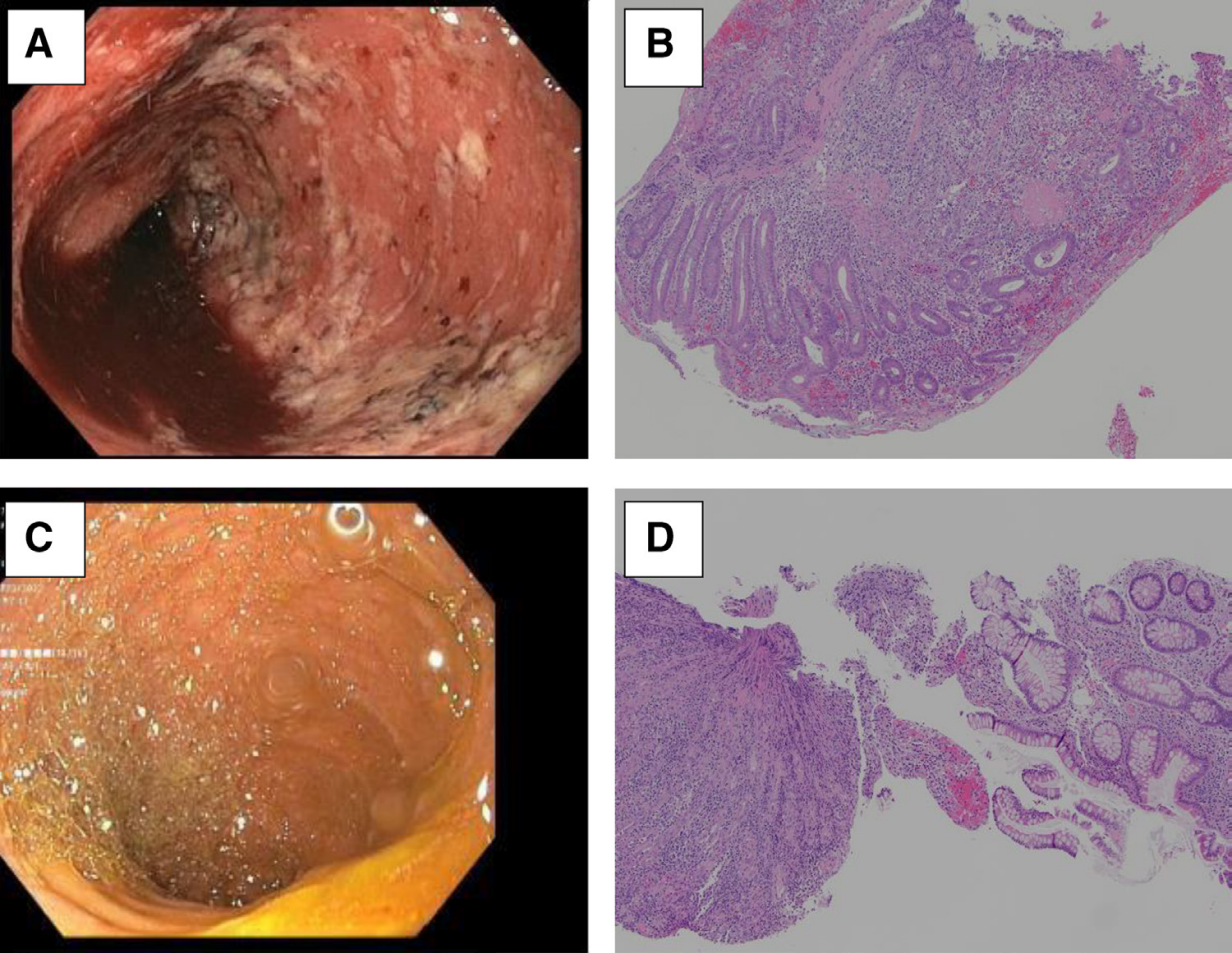
(A) Initial ileoscopy image—diffuse inflammation characterized by erythema, exudate, and friability. (B) Initial ileal histopathology—severe active ileitis, erosion, and focal crypt irregularity (magnification 100×). Repeat ileoscopy image– normal mucosa. (D) Repeat ileal histopathology—nonspecific changes including patchy lamina propria lymphoplasma cell infiltrate, eosinophilia, and spotty glandular and intraepithelial lymphocytosis (magnification 100×).

A 16-year-old male was initially diagnosed with moderate to severe ulcerative pancolitis (Mayo endoscopic score 2–3) (8). Initial upper endoscopy and baseline magnetic resonance enterography (MRE) were normal. He was started on sulfasalazine and achieved clinical remission as defined by symptom resolution. One year later, he developed worsening diarrhea, hematochezia, and abdominal pain and was treated for active UC with oral corticosteroids, then infliximab. Despite a trial of intravenous steroids and infliximab dose optimization, his pediatric UC activity index (PUCAI) was consistent with acute severe colitis (>80). He ultimately required a laparoscopic total colectomy with end ileostomy. Histopathology of the surgical specimen was consistent with the prior diagnosis of UC: continuous, diffuse chronic active colitis with focal ulceration, nonspecific focally active inflammatory changes without evidence of chronicity in the ileum, and no evidence of viral inclusions or granulomas in the ileum. On postoperative day 4, the patient developed severe nausea and pain around his stoma. He then developed fevers, worsening abdominal pain and a sudden increase in ostomy output ranging from 4 to 6 L/day. He had a thorough infectious evaluation, including testing for *Clostridium difficile*, which was negative (Table 1). Abdominal computed tomography with intravenous and enteral contrast showed progressive moderate diffuse distention of his small bowel loops with no clear evidence of stricture or obstruction. Following review of this imaging and a normal digital examination of the stoma, our pediatric surgery colleagues did not think functional or mechanical dysfunction of the stoma were likely. On postoperative day 13, upper endoscopy and ileoscopy revealed erythema of the duodenal mucosa and diffuse severe ileal inflammatory changes with friability and bleeding (Fig. 1A). Histology showed chronic active duodenitis and severe active ileitis with erosion and focal crypt irregularity (Fig. 1B). He was started on intravenous corticosteroids (60 mg) daily without significant improvement over 2 weeks. As his corticosteroid dose was tapered by 5 mg/day each week, ostomy output decreased, and abdominal pain and distension improved. Four weeks later (6 weeks postoperatively), repeat upper endoscopy and ileoscopy demonstrated resolution of his duodenitis and ileitis grossly (Fig. 1C). Histology showed marked improvement in inflammatory changes, with only mild variable and nonspecific findings in the duodenum and ileum (Fig. 1D). At follow-up visit with his primary gastroenterologist 1 month after discharge, he had ongoing symptom improvement and weight gain.

**TABLE 1.** Infectious disease evaluation

Blood
Severe acute respiratory syndrome (SARS) COVID antibody
Adenovirus (AdV) PCR
Cytomegalovirus PCR
Herpes simplex virus (HSV) PCR
Histoplasmosis antigen
Beta 1.3 D-glucan
Ileal Tissue
Epstein Barr virus encoded RNA stain
AdV PCR
HSV PCR
Film array viral panel*
Stool
Gastrointestinal film array panel†
Urine
Histoplasmosis antigen

*BioFire Film Array Respiratory Panel, Biomerieux—tests for 22 bacterial and viral respiratory pathogens.

†BioFire Film Array Gastrointestinal Panel, Biomerieux, RP2.1 software—tests for 22 bacterial, viral, and parasitic enteric pathogens including *C. difficile*.

## DISCUSSION

This is the first published case of a pediatric patient with PCE, an entity previously only described in adults. PCE may be difficult to diagnose; in patients initially diagnosed with UC who develop small bowel inflammation following colectomy, the concern is often misdiagnosed Crohn’s disease (6, 7). In the immediate postoperative period, one must also consider surgical complications such as wound infection, anastomotic leak, or ischemia, functional and mechanical stoma complications including stricture and prolapse, and bacterial, viral, and fungal infectious complications including *Clostridium difficile* infection (Table 1) (3, 7). However, with sudden abrupt onset of worsening pain and ostomy output, and histology consistent with diffuse small bowel inflammation, PCE should be considered in pediatrics as well. One study reported that diagnosis of PCE should be based on the following: underlying diagnosis of UC, characteristic symptoms, findings on endoscopy consistent with the diagnosis, the ability to exclude Crohn’s disease with histology and complications consistent with Crohn’s disease, and response to treatment such as corticosteroids (5).

Case reports in adults have shown improvement with use of corticosteroids and anti-TNFα medications in the acute setting with variable need for long-term treatment. This is likely due to the possible pathogenesis of PCE related to transient abnormal T-helper cell ratios and increased cytokine production (4). Our patient did not require further immunosuppressive therapy and ultimately had improvement in symptoms with corticosteroid taper and supportive care. In a study of over 800 adult patients with UC status postcolectomy, 7 developed PCE, 3 of whom did not respond to corticosteroids (6). There is no consensus on optimal treatment for PCE, and further studies are needed to understand the natural history and optimal management of pediatric postcolectomy enteritis.

In summary, although PCE has not previously been described in a pediatric patient with UC, it should be considered in those with acute onset of symptoms including increased ostomy output and abdominal pain following colectomy. Thorough evaluation for more common etiologies such as surgical complications, infection, or misdiagnosed Crohn’s disease should be performed.

## ACKNOWLEDGMENTS

The patient provided informed assent and his legal guardian provided informed consent for publication of the details of this case.

## References

[1] Ford AC Moayyedi P Hanauer SB. Ulcerative colitis. BMJ. 2013;346:f432. 23386404 10.1136/bmj.f432

[2] Dignass A Lindsay JO Sturm A. Second European evidence-based consensus on the diagnosis and management of ulcerative colitis part 2: current management. J Crohns Colitis. 2012;6:991–1030. 23040451 10.1016/j.crohns.2012.09.002

[3] Ghouri YA Tahan V Shen B. Secondary causes of inflammatory bowel diseases. World J Gastroenterol. 2020;26:3998–4017. 32821067 10.3748/wjg.v26.i28.3998 PMC7403802

[4] Akitake R Nakase H Tamaoki M. Modulation of Th1/Th2 balance by infliximab rescues postoperative occurrence of small-intestinal inflammation associated with ulcerative colitis. Dig Dis Sci. 2010;55:1781–1784. 19672713 10.1007/s10620-009-0910-5

[5] Hoentjen F Hanauer SB Hart J. Long-term treatment of patients with a history of ulcerative colitis who develop gastritis and pan-enteritis after colectomy. J Clin Gastroenterol. 2013;47:52–57. 22858512 10.1097/MCG.0b013e3182582c1d PMC3874322

[6] Horio Y Uchino M Hori K. Clinical features and therapeutic outcomes of post-colectomy enteritis with ulcerative colitis. J Anus Rectum Colon. 2021;5:405–413. 34746505 10.23922/jarc.2021-031 PMC8553349

[7] Kohyama A Watanabe K Sugita A. Ulcerative colitis-related severe enteritis: an infrequent but serious complication after colectomy. J Gastroenterol. 2021;56:240–249. 33155079 10.1007/s00535-020-01742-3

[8] Schroeder KW Tremaine WJ Ilstrup DM. Coated oral 5-aminosalicylic acid therapy for mildly to moderately active ulcerative colitis. A randomized study. N Engl J Med. 1987;317:1625–1629. 3317057 10.1056/NEJM198712243172603

